# Facile Synthesis of Non-Graphitizable Polypyrrole-Derived Carbon/Carbon Nanotubes for Lithium-ion Batteries

**DOI:** 10.1038/srep19317

**Published:** 2016-01-14

**Authors:** Bo Jin, Fan Gao, Yong-Fu Zhu, Xing-You Lang, Gao-Feng Han, Wang Gao, Zi Wen, Ming Zhao, Jian-Chen Li, Qing Jiang

**Affiliations:** 1 Key Laboratory of Automobile Materials, Ministry of Education, and College of Materials Science and Engineering, Jilin University, Changchun, 130022, China

## Abstract

Graphite is usually used as an anode material in the commercial lithium ion batteries (LIBs). The relatively low lithium storage capacity of 372 mAh g^–1^ and the confined rate capability however limit its large-scale applications in electrical vehicles and hybrid electrical vehicles. As results, exploring novel carbon-based anode materials with improved reversible capacity for high-energy-density LIBs is urgent task. Herein we present TNGC/MWCNTs by synthesizing tubular polypyrrole (T-PPy) via a self-assembly process, then carbonizing T-PPy at 900 °C under an argon atmosphere (TNGC for short) and finally mixing TNGC with multi-walled carbon nanotubes (MWCNTs). As for TNGC/MWCNTs, the discharge capacity of 561 mAh g^−1^ is maintained after 100 cycles at a current density of 100 mA g^−1^. Electrochemical results demonstrate that TNGC/MWCNTs can be considered as promising anode materials for high-energy-density LIBs.

Lithium-ion batteries (LIBs) as the most important power sources are in a variety of applications such as mobile phones, laptop computers, digital cameras, electrical vehicles (EVs) and hybrid electrical vehicles (HEVs) because they offer relatively high specific capacity compared with conventional batteries^1^,^2^,^3^,^4^. In the commercial LIBs, graphite is usually utilized as an anode material, which is attributed to its easy Li^+^ insertion/de-insertion, long cycling life, increasing supply of raw materials and low cost^5^,^6^,^7^,^8^,^9^. However, the relatively low lithium storage capacity of graphite is limited to be 372 mAh g^−1^, assuming a reaction of Li with C to LiC_6_^10^,^11^. Also, the rate capability induced by its low Li diffusion coefficient is limited. These restrict its large-scale applications in EVs and HEVs. Therefore, it is very important to explore novel carbon-based anode materials and further to improve the reversible capacity of high-energy-density LIBs.

Recently, non-graphitizable polypyrrole (PPy)-derived carbon has been investigated as an anode material for LIBs^12^,^13^,^14^,^15^. Qie *et al*. report a strategy to synthesize porous carbon that shows a nanostructure with large surface area and high-level nitrogen doping. After carbonization-activation of PPy with KOH, carbon nanofiber webs (CNFWs) doped with nitrogen were obtained. Benefiting from the unique porous nanostructure and high-level N doping, CNFWs exhibit superhigh capacity and excellent rate capability, delivering a reversible capacity as high as 943 mAh g^−1^ at a current density of 2 A g^−1^ even after 600 cycles^12^. Zhou *et al*. prepared high yield porous carbon with a seaweed-like porous morphology via the chemical oxidative polymerization of pyrrole and subsequent decomposition of PPy nanowires with KOH activation. Owing to the unique microstructure, the seaweed-like porous carbon exhibits satisfactory electrochemical properties, such as high reversible capacity, good rate performance and stable cyclability^13^. Wu *et al*. synthesized non-graphitic carbon nanotubes (NGCNTs) by carbonization of PPy nanotubes precursor prepared via a self-assembly process. NGCNTs deliver an initial reversible capacity of 635.7 mAh g^−1^ at current density of 100 mA g^−1^ with a high capacity retention ratio of 85.7% after 150 cycles. Even up to 4 A g^−1^, the reversible capacity of NGCNTs remains in 280.1 mAh g^−1^. The improved performance of NGCNTs is attributed to the non-graphitic form, tubular morphology and cross-linked conducting networks^14^. In summary, the non-graphitizable PPy-derived carbon manifests satisfactory electrochemical performance; however, its reversible capacity, rate performance and cycling stability still need to be greatly improved for high-energy-density LIBs. Both nitrogen doping and carbon coating are expected to be effective strategies to resolve these problems where nitrogen doping is complicated and time-consuming while carbon nanotube coating is relatively facile. Multi-walled carbon nanotubes (MWCNTs) as conductive agent have been widely utilized to improve the electrochemical performance of the electrode materials due to its excellent conductivity and mechanical properties^16^,^17^,^18^,^19^,^20^,^21^,^22^,^23^,^24^,^25^,^26^. In our early papers, MWCNTs indeed improve the electrochemical performance of LiFePO_4_ and elemental sulfur^27^,^28^. Based on above reasons, MWCNTs in this study were added to activate the electrochemical performance of the non-graphitizable PPy-derived carbon.

In this work, we fabricate TNGC/MWCNTs by synthesizing tubular polypyrrole (T-PPy) via a self-assembly process, then carbonizing T-PPy at 900 °C under an argon atmosphere (TNGC) and finally mixing TNGC with MWCNTs. This synthesis process of the as-prepared carbon is simple and would be a facile way to produce more carbon on large scales. The electrochemical performance of TNGC/MWCNTs was evaluated by cyclic voltammogram and galvanostatic discharge/charge testing.

## Results

**Figure 1:**
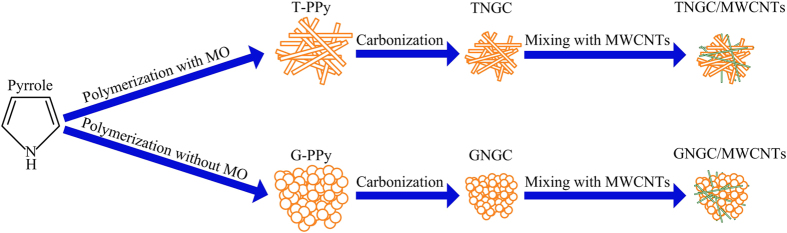
The schematic illustration for the synthesis of GNGC/MWCNTs and TNGC/MWCNTs. The orange rectangle represents T-PPy and TNGC, the green rectangle represents MWCNTs, and the orange circle represents G-PPy and GNGC.

**Figure 2:**
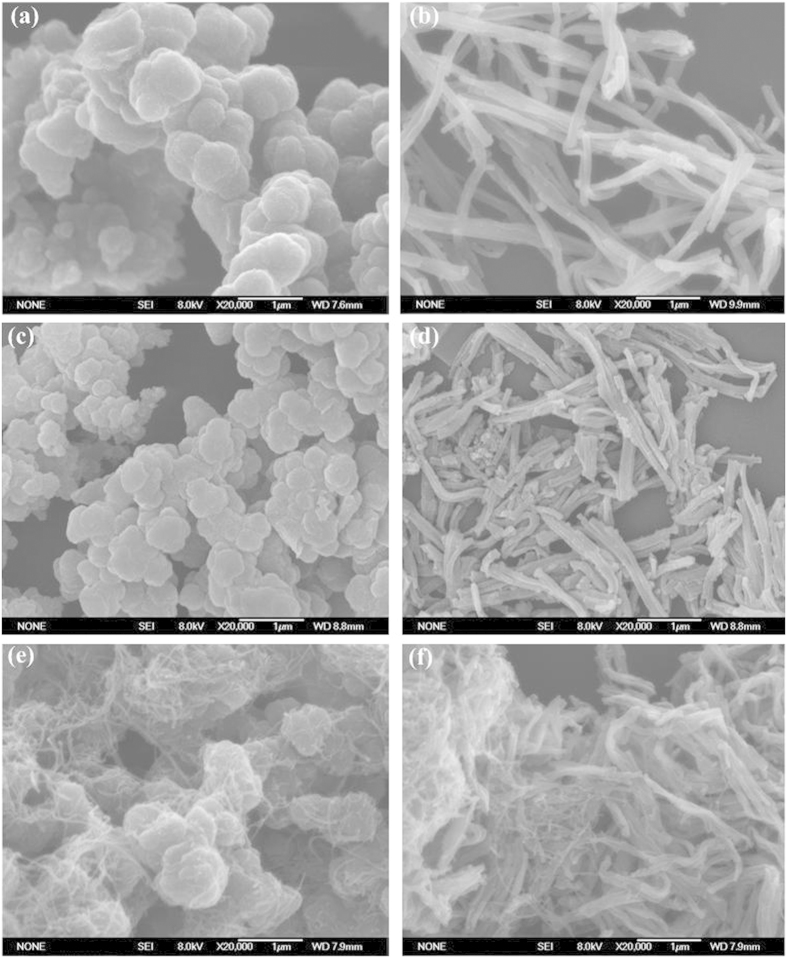
Microstructure characterization. FESEM images of (**a**) G-PPy, (**b**) T-PPy, (**c**) GNGC, (**d**) TNGC, (**e**) GNGC/MWCNTs and (**f**) TNGC/MWCNTs.

**Figure 3:**
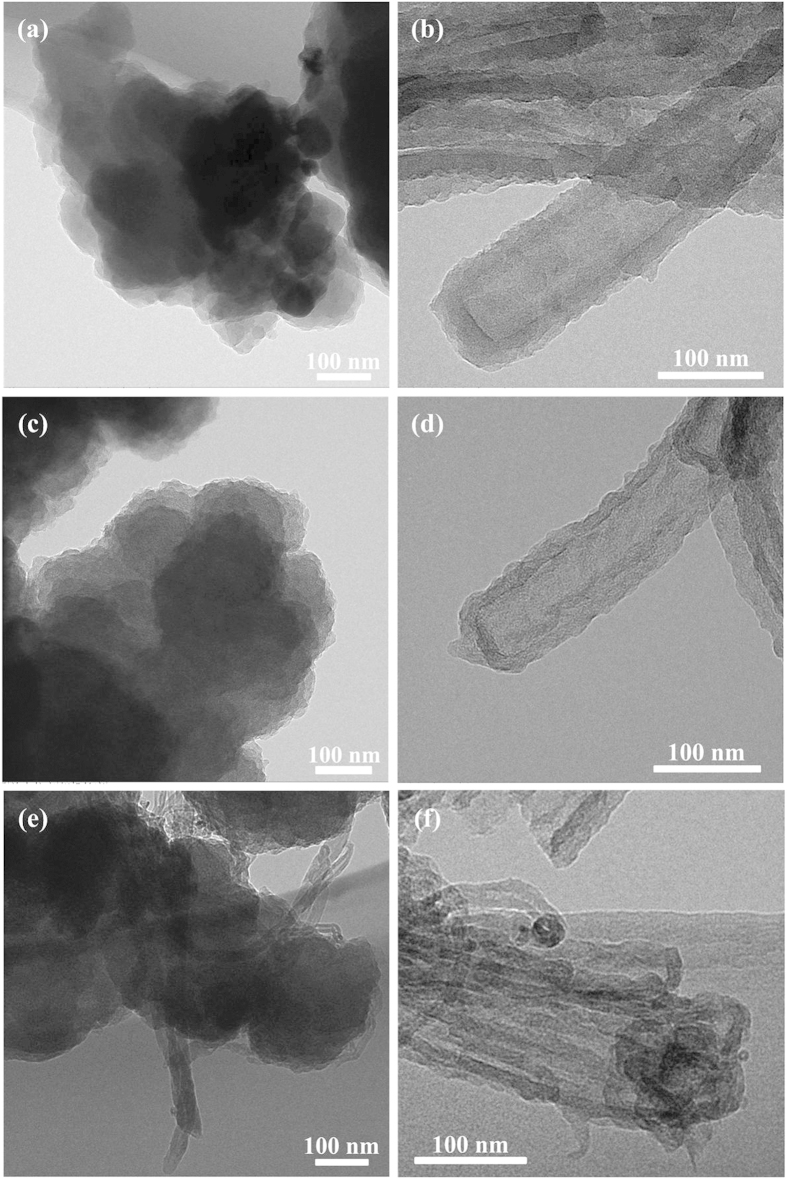
Microstructure characterization. TEM images of (**a**) G-PPy, (**b**) T-PPy, (**c**) GNGC, (**d**) TNGC, (**e**) GNGC/MWCNTs and (**f**) TNGC/MWCNTs.

A typical synthesis of T-PPy was a self-assembly process in the presence of methyl orange (MO). For comparison, granular polypyrrole (G-PPy) was synthesized under the same condition but without adding MO. The as-obtained T-PPy and G-PPy were further carbonized to fabricate non-graphitizable carbon (NGC) in a quartz tubular furnace under an argon atmosphere. Two types of the NGC fabricated by carbonizing G-PPy and T-PPy were denoted as GNGC and TNGC, respectively. GNGC/MWCNTs and TNGC/MWCNTs were synthesized by mixing GNGC or TNGC with 5 wt% MWCNTs, respectively. The synthesis route for GNGC/MWCNTs and TNGC/MWCNTs is shown in Fig. 1. FESEM and TEM were used to investigate the morphologies and microstructures of G-PPy, T-PPy, GNGC, TNGC, GNGC/MWCNTs and TNGC/MWCNTs. The FESEM and TEM images are shown in Figs 2 and 3, respectively. As shown in Fig. 2a, the morphology of PPy is granular without adding MO, and the particle size of G-PPy is in the range of ~0.6–1 μm in diameter. As shown in Fig. 2c, the morphology of GNGC still preserves granular microstructure after the G-PPy was carbonized into GNGC, and the particle size of GNGC ranges from ~400 to ~600 nm in diameter. Figure 2b gives homogenous morphology of tubular microstructure of PPy with outer diameter of ~100–200 nm in the presence of MO. The formation mechanism of T-PPy is the same as the previous works^14^,^29^,^30^. In the presence of MO, FeCl_3_ and MO can form a fibrillar complex template. The pyrrole monomers were polymerized on the surface of the fibrillar complex template by the oxidation function of Fe^3+^ ions. In the meantime the reduction of Fe^3+^ to Fe^2+^ leads to the automatic degradation of the template itself and T-PPy was finally formed. The morphology of PPy retains tubular microstructure after carbonization as shown in Fig. 2d, and the outer diameter of TNGC is ca. 120 nm. It is obvious that the carbonization of G-PPy or that of T-PPy does not change its initial morphology. Thus, the method used here is propitious to the design of nanostructured carbon with required morphology. As shown in Figs 2(e,f) and 3, the added MWCNTs intertwine with GNGC or TNGC together to form a three-dimensional network, and the well-dispersed MWCNTs may provide access to the electron transference, leading to the improvement in the electrochemical performance of GNGC/MWCNTs and TNGC/MWCNTs.

**Figure 4:**
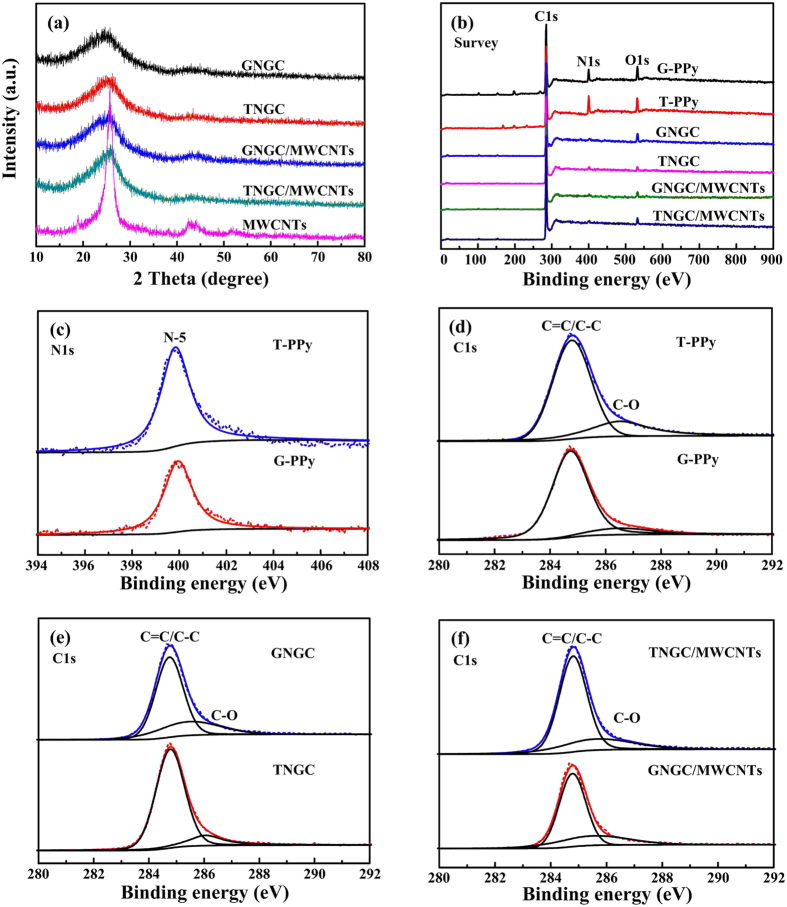
Microstructure characterization. (**a**) XRD patterns of GNGC, TNGC, GNGC/MWCNTs, TNGC/MWCNTs and MWCNTs. XPS spectra of G-PPy, T-PPy, GNGC, TNGC, GNGC/MWCNTs and TNGC/MWCNTs: (**b**) survey, (**c**) N1s, (**d–f**) C1s.

XRD patterns of GNGC, TNGC, GNGC/MWCNTs and TNGC/MWCNTs are shown in Fig. 4a. As a comparison, XRD pattern of the commercial MWCNTs with well graphite microstructure is also displayed in Fig. 4a where all five samples contain the diffraction peak at ~25.76°, corresponding to [002] of the graphite microstructure. The diffraction peaks in GNGC, TNGC, GNGC/MWCNTs and TNGC/MWCNTs are weak and broad compared to the sharp and narrow diffraction peak in MWCNTs, demonstrating their low crystallinity and disordered amorphous microstructures^14^,^29^,^31^,^32^. As can be observed in Fig. 4a, in GNGC/MWCNTs and TNGC/MWCNTs, there is no obvious MWCNTs diffraction peak which is ascribed to [101] of the graphite microstructure at ~43.55° due to its low content. It is evident that the added MWCNTs do not change the morphologies and the disordered amorphous microstructures of GNGC and TNGC. XPS analysis was carried out to study the elemental composition, purity and bonding nature existed in G-PPy, T-PPy, GNGC, TNGC, GNGC/MWCNTs and TNGC/MWCNTs. The XPS survey spectra of the six samples (Fig. 4b) display the existence of C, O and N atoms (only for G-PPy and T-PPy) without any impurities, indicating that both G-PPy and T-PPy have been successfully carbonized. As shown in Fig. 4c, both G-PPy and T-PPy exhibit only one peak at 399.9 eV (N-5), ascribing to N atoms within the pentagonal pyrrole ring of PPy. The N-5 corresponds to pyrrolic N in a five-membered ring that is associated with phenolic or carbonyl group on the neighbor C atom of the ring^33^. Figure 4(d–f) indicate that the fitted C1s spectra of the six samples show two obvious peaks located at 284.7 and 286.5 eV, which can be attributed to sp^2^ C=C/sp^3^ C–C and C–O bonds, respectively.

**Figure 5:**
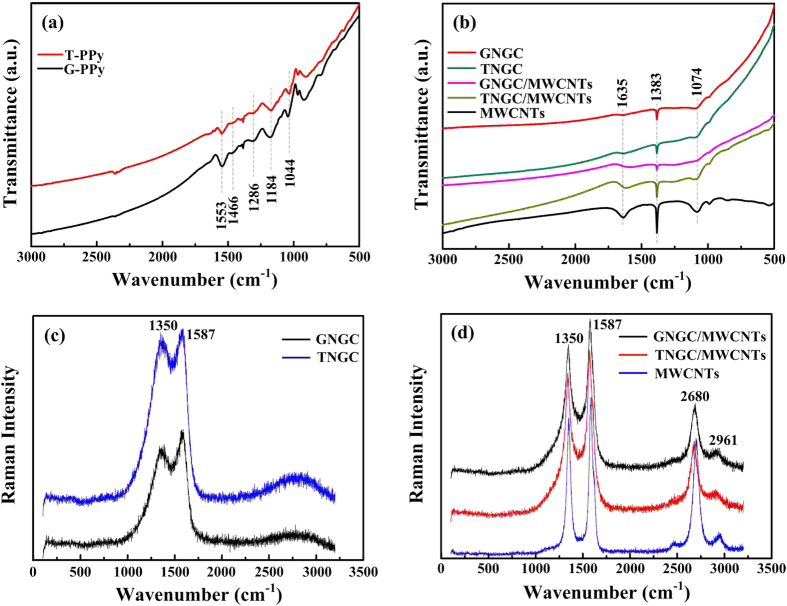
Microstructure characterization. FTIR spectra of the seven samples: (**a**) G-PPy and T-PPy, (**b**) GNGC, TNGC, GNGC/MWCNTs, TNGC/MWCNTs and MWCNTs. Raman spectra of the five samples: (**c**) GNGC and TNGC, (**d**) GNGC/MWCNTs, TNGC/MWCNTs and MWCNTs.

FTIR spectroscopy was used to determine the microstructure. FTIR spectra of G-PPy, T-PPy, GNGC, TNGC, GNGC/MWCNTs, TNGC/MWCNTs and MWCNTs were recorded in the region of 500–3000 cm^−1^ on a Bruker TENSOR 27 spectrometer and the results were present in Fig. 5(a,b). As shown in Fig. 5a, G-PPy and T-PPy present almost identical spectra, which is consistent with the references^17^,^34^,^35^. In the FTIR spectra, the characteristic bands at ~1553 and ~1466 cm^−1^ can be assigned to the C=C and C–N fundamental vibrations of pyrrole ring, the characteristic peaks at ~1286 and ~1044 cm^−1^ can be ascribed to the =C–H in-plane vibration and in-plane N–H deformation of PPy chain, respectively, and the characteristic band at ~1184 cm^−1^ corresponds to the C–N–C stretching vibration in the polaron microstructure. These results imply that G-PPy and T-PPy have been successfully received. As shown in Fig. 5b, the characteristic peaks of G-PPy and T-PPy disappear, both GNGC and TNGC display broader and overlapping bands due to the strong absorption of carbon^36^, and exhibit the characteristic peaks at ~1635 cm^−1^ attributed to the C=C stretching vibration in aromatic ring, indicating that both G-PPy and T-PPy are carbonized into carbon-based materials. The characteristic peaks at ~1635 cm^−1^ become stronger due to the existence of MWCNTs in GNGC/MWCNTs and TNGC/MWCNTs. As shown in Fig. 5b, the characteristic peaks at ~1383 and ~1074 cm^−1^ correspond to the C–OH bending deformation vibration and the C–O–C stretching vibration, respectively.

The microstructures of GNGC, TNGC, GNGC/MWCNTs, TNGC/MWCNTs and MWCNTs have also been determined by Raman spectra [Fig. 5(c,d)]. In all cases, typical D (sp^3^–type) and G (sp^2^–type) bands of carbon are located at ~1350 and ~1587 cm^−1^, respectively. The G band at ~1587 cm^−1^ is assigned to the E_2g_ graphite mode, and D band at ~1350 cm^−1^ is associated to A_1g_ mode, which is related to the breakage of symmetry occurring at the edges of graphite sheets^37^. The bands at ~1587 and ~1350 cm^−1^ are originated from graphitic and amorphous forms, respectively. The characteristic peaks at ~1350, ~1587 and ~2680 cm^−1^ confirm the existence of MWCNTs in GNGC/MWCNTs and TNGC/MWCNTs. Because of the existence of the MWCNTs, the characteristic bands at ~1350 and ~1587 cm^−1^ become strong and relatively narrow compared to those of GNGC and TNGC. Additional bands at ~2680 and ~2961 cm^−1^ are observed. These are namely 2D and D + G bands, which are attributed to the overtones or the combinations of the first-order phonon modes^38^,^39^. The presence of both D band and G band demonstrates more disordered amorphous microstructures of GNGC, TNGC, GNGC/MWCNTs and TNGC/MWCNTs, which is in accord with XRD results in Fig. 4a. Similar results are also observed with the mesoporous carbon obtained at 550 °C^40^.

**Figure 6:**
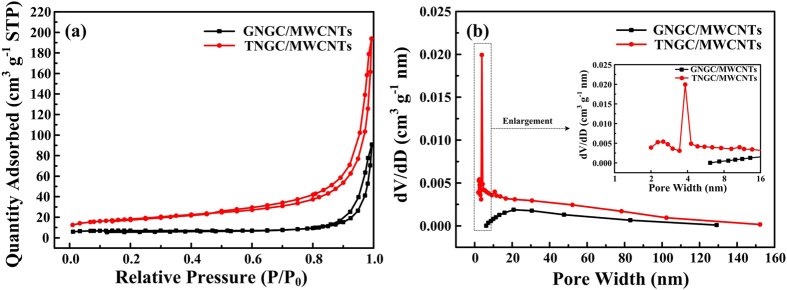
Microstructure characterization. (**a**) Nitrogen adsorption/desorption isotherms and (**b**) corresponding pore size distribution curves of GNGC/MWCNTs and TNGC/MWCNTs. The insets in Fig. 5b manifest the enlargement of pore size distribution.

Furthermore, the specific surface areas and porous structures of GNGC/MWCNTs and TNGC/MWCNTs were investigated by N_2_ adsorption/desorption analysis at 77 K. Figure 6 displays the adsorption/desorption isotherms and corresponding pore size distribution curves of the two samples. All two samples exhibit type II adsorption/desorption isotherms. TNGC/MWCNTs with tubular microstructure display the BET surface area of 62.5 m^2 ^g^−1^. However, the BET surface area of GNGC/MWCNTs is only 20.9 m^2 ^g^−1^. Thus, TNGC/MWCNTs possess a larger surface area than another, and is larger than that of the commercial graphite (4.6 m^2 ^g^−1^) and beneficial to the electrochemical reaction. TNGC/MWCNTs with tubular microstructure manifest a pore volume of 0.16 cm^3 ^g^−1^, which is 2.5 times of GNGC/MWCNTs (0.064 cm^3 ^g^−1^). As shown in Fig. 6b, the pore size of the TNGC/MWCNTs with tubular microstructure centralizes on two areas with peak values of ~2.5 and ~4 nm, which can be attributed to the inherent mesopores of the materials. This mesoporous microstructure provides low-resistance channels and shorter diffusion routes for the ions through the porous microstructure. Such porous tubular microstructure can facilitate the percolation of the electrolyte into active particles, create more convenient pathways for electrolyte ion remotion, and provide electroactive sites for fast energy storage at large current densities. Such performance is very vital for LIBs electrode materials with a large specific capacity and high-rate charge/discharge ability.

**Figure 7:**
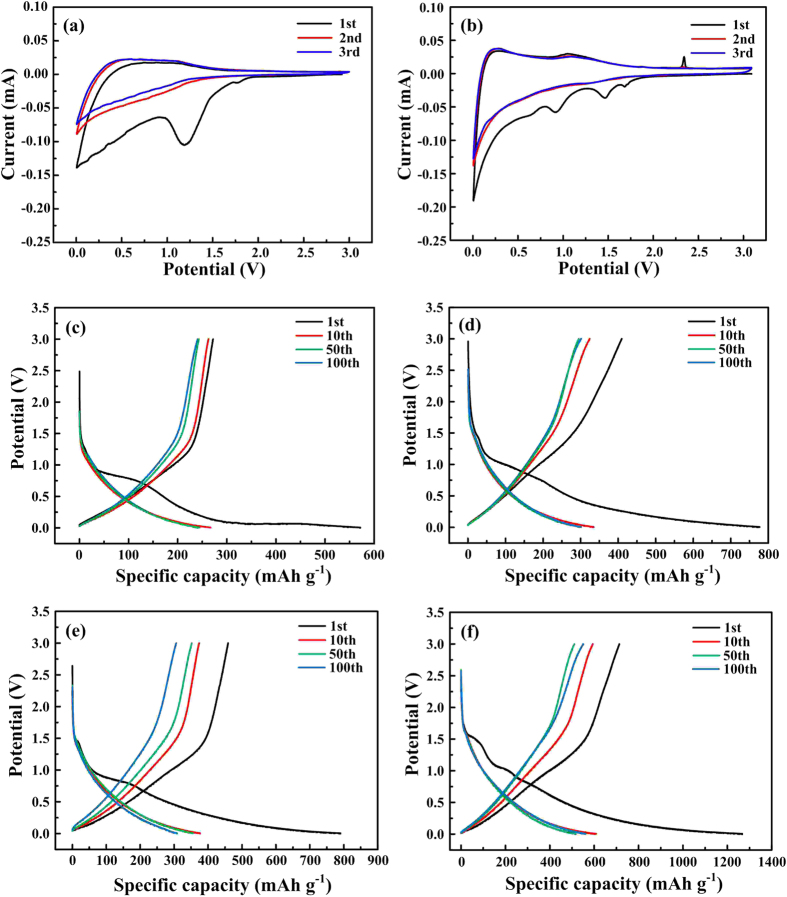
Electrochemical performance. Cyclic voltammograms of (**a**) GNGC/MWCNTs and (**b**) TNGC/MWCNTs at a scan rate of 0.1 mV s^−1^ from 0.005 to 3.0 V. Typical discharge/charge curves of (**c**) GNGC, (**d**) TNGC, (**e**) GNGC/MWCNTs and (**f**) TNGC/MWCNTs after different cycles at a current density of 100 mA g^−1^ in the voltage range of 0.005–3.0 V.

To better understand the lithium intercalation/de-intercalation mechanism, CV measurements of GNGC/MWCNTs and TNGC/MWCNTs at a scan rate of 0.1 mV s^−1^ from 0.005 to 3.0 V were performed [Fig. 7 (a,b)]. In the first cathodic scan, all two samples display an irreversible reduction peak at ~1 V, which is attributed to the formation of solid electrolyte interphase (SEI) layer on the carbon electrode due to decomposition of the electrolyte on the surface of the electrode^12^,^41^,^42^,^43^,^44^. TNGC/MWCNTs appear to undergo other reactions assigned to the protuberant reduction peaks at ~1.5 and ~1.7 V, and also the protuberant reduction peak at ~1.7 V for GNGC/MWCNTs. This result may come from the irreversible insertion of Li^+^ into the interfacial storage sites. In the subsequent cycles, the reduction peak at ~1 V disappears, implying the loss of some irreversible lithium storage sites during the first cycle^45^. The absent protuberant reduction peaks at ~1.5 and ~1.7 V predicate the end of other reactions. A reduction peak starting from 0.5 to 0.005 V corresponds to Li^+^ intercalating into TNGC/MWCNTs or GNGC/MWCNTs. In the first anodic scan, an oxidation peak with a broad shoulder is observed at ~0.1–1.5 V ascribing to Li^+^ extraction from TNGC/MWCNTs or GNGC/MWCNTs. As for TNGC/MWCNTs, there seems another reaction ascribed to an oxidation peak at ~2.3 V, which may correspond to Li^+^ extraction from the interfacial storage sites. In the subsequent cycles, the oxidation peak at ~2.3 V disappears, denoting the end of the other reaction. During the subsequent CV scans, the almost overlap of the second and the third cycles for TNGC/MWCNTs demonstrates that TNGC/MWCNTs possess more excellent cycle stability than GNGC/MWCNTs.

Typical discharge/charge curves of GNGC, TNGC, GNGC/MWCNTs and TNGC/MWCNTs after different cycles at a current density of 100 mA g^−1^ in the voltage range of 0.005–3.0 V are shown in Fig. 7(c–f) where all four samples display sloping curves without obvious voltage plateaus during the discharge/charge process, which are typical features of the carbonaceous materials and consistent with previous literatures^40^,^44^,^45^,^46^,^47^,^48^,^49^,^50^. The initial discharge and charge capacities of TNGC are 777.2 and 409.6 mAh g^−1^, respectively, and the initial Columbic efficiency (ICE) is 52.7%. As for GNGC, it can only deliver the initial discharge and charge capacities of 572 and 272.2 mAh g^−1^, respectively, with ICE of 47.6%. However, the initial discharge and charge capacities of TNGC/MWCNTs (1267.3 and 713.5 mAh g^−1^) and GNGC/MWCNTs (790.2 and 459.2 mAh g^−1^) are superior to those of GNGC and TNGC. The ICE of TNGC/MWCNTs and GNGC/MWCNTs is 56.3% and 58.1%, respectively, and comparable to those of GNGC and TNGC. The initial capacity loss and relatively low ICE of the four samples may be ascribed to the formation of SEI layer on the carbon electrode due to the decomposition of electrolyte on the surface of the electrode^12^,^41^,^42^,^43^,^44^.

**Figure 8:**
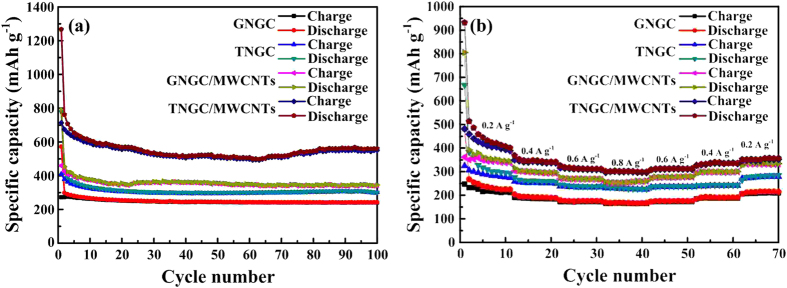
Electrochemical performance. (**a**) Cycling performance of GNGC, TNGC, GNGC/MWCNTs and TNGC/MWCNTs at a current density of 100 mA g^−1^ in the voltage range of 0.005–3.0 V. (**b**) Rate performance of GNGC, TNGC, GNGC/MWCNTs and TNGC/MWCNTs at different current densities.

To evaluate the cycle stability of GNGC, TNGC, GNGC/MWCNTs and TNGC/MWCNTs, the electrodes were cycled in the voltage range of 0.005–3.0 V at a current density of 100 mA g^−1^, and the results are shown in Fig. 8a. All four samples show the stable cycle life up to 100 cycles. Among them, TNGC/MWCNTs show excellent cycle stability with a discharge capacity of 561 mAh g^−1^ even after 100 cycles, comparable to the reported values (Table 1), which is higher than the theoretical capacity of the commercial graphite (372 mAh g^−1^), whereas the discharge capacities of GNGC, TNGC and GNGC/MWCNTs are only 242.4, 301.2 and 344.8 mAh g^−1^ upon 100 cycles, respectively. The excellent cycle stability and the improved lithium storage capacity of TNGC/MWCNTs are consistent with the CV and the typical discharge/charge test results in Fig. 7, which can be explained as follows. First, MWCNTs act as conductive agent to provide a fast path for electron transportation. Second, TNGC/MWCNTs with the porous tubular microstructure possess little volume bulge during discharge/charge process. In the meantime the unique porous tubular microstructure can favor the electrolyte percolation and shorten lithium-ion diffusion pathway, and lead to sufficient electrode/electrolyte interface to absorb Li^+^ and promote reversibility of lithium insertion into/extraction from TNGC^14^,^51^,^52^. Third, both MWCNTs and TNGC possess tubular microstructures, and can easily form cross-linked conducting networks in the electrode and serve as shorter and more continuous channels for electron transportation, and thus reduce the resistance of the electrode^44^.

Rate performance of GNGC, TNGC, GNGC/MWCNTs and TNGC/MWCNTs was examined via galvanostatic cycle tests, as shown in Fig. 8b. For the testing, all four cells were cycled for 10 cycles at various current densities from 0.2 to 0.8 A g^−1^, and then cycled for 10 cycles when the current densities were varied from 0.8 to 0.2 A g^−1^. As for the four cells, the rate capability increases in the following order: GNGC < TNGC < GNGC/MWCNTs < TNGC/MWCNTs. The discharge capacity of TNGC/MWCNTs is stabilized at 407.2 mAh g^−1^ after 10 cycles at a current density of 0.2 A g^−1^. With ascending discharge/charge current densities to 0.4, 0.6 and 0.8 A g^−1^, the discharge capacities are 341.8, 311.7 and 299.2 mAh g^−1^, respectively. When the discharge/charge current densities are turned back to 0.6, 0.4 and 0.2 A g^−1^, the discharge capacities are recovered to 313, 335.4 and 357 mAh g^−1^, respectively, indicating very stable cycling performance. TNGC/MWCNTs manifest the superior rate capability, which is attributed to both MWCNTs as conductive agent and the porous tubular microstructure of TNGC.

In summary, we have successfully developed TNGC/MWCNTs by synthesizing T-PPy via a self-assembly process, then carbonizing T-PPy at 900 °C under an argon atmosphere and finally mixing TNGC with MWCNTs. It is found that MO is a key factor in controlling the morphology of PPy. The results indicate that the electrochemical performance of the obtained TNGC/MWCNTs is excellent, which can be attributed to both MWCNTs as conductive agent and porous tubular microstructure of TNGC. The synergistic effects of MWCNTs and the porous tubular microstructure of TNGC result in excellent electronic conductivity, little volume bulge during discharge/charge process, easy electrolyte percolation, short lithium-ion diffusion pathway, sufficient electrode/electrolyte interface to absorb Li^+^ and excellent reversibility of lithium insertion/extraction. Such TNGC/MWCNTs have the great promise to be anode materials for energy storage applications.

## Methods

### Fabrication of GNGC, TNGC, GNGC/MWCNTs and TNGC/MWCNTs

A typical synthesis of T-PPy was a self-assembly process in the presence of methyl orange (MO). 51.1 mg MO (0.15 mmol, Aladdin Industrial Corporation) was firstly dissolved in 30 ml deionized water under magnetically stirring. Then 405 mg FeCl_3_·6H_2_O (1.5 mmol, Sinopharm Chemical Reagent Co. Ltd.) was added. After FeCl_3_·6H_2_O was dissolved, pyrrole monomer (105 μL, 1.5 mmol, Aladdin Industrial Corporation) was added into the solution. The mixture was stirred at room temperature for 24 h. PPy precipitate was washed to neutral using deionized water and ethanol several times, and then dried under vacuum for 24 h at 60 °C to get T-PPy. For comparison, granular polypyrrole (G-PPy) was synthesized under the same condition but without adding MO. The as-obtained T-PPy and G-PPy were further carbonized to fabricate non-graphitizable carbon (NGC) in a quartz tubular furnace under an argon atmosphere. The samples were heated at 900 °C for 3 h and then naturally cooled down to room temperature. Two types of the NGC fabricated by carbonizing G-PPy and T-PPy were denoted as GNGC and TNGC, respectively. GNGC/MWCNTs and TNGC/MWCNTs were synthesized by mixing GNGC or TNGC with 5 wt% MWCNTs, respectively. The as-obtained NGC and MWCNTs were added into 70 mL ethanol, and ultrasonically mixed for 0.5 h. After that, the mixture was kept under magnetically stirring for 6 h, and finally dried under vacuum for 24 h at 60 °C to get the non-graphitizable PPy-derived carbon/MWCNTs.

### Structural characterization

The crystalline phases were identified with X-ray diffraction (XRD, Dmax/2500PC, Rigaku, Japan) with Cu Kα radiation (λ = 1.5406 Å). Powder morphologies were observed by field emission scanning electron microscope (FESEM, JSM-6700F, Japan) and transmission electron microscope (TEM, JEM-2100F, Japan) with an acceleration voltage of 200 kV. Fourier transform infrared spectra were recorded with a Bruker TENSOR 27 spectrometer. Raman spectra were measured with a Renishaw micro-Raman spectrometer using 532 nm argon-ion laser as excitation radiation source. The surface chemistry of the samples was investigated by X-ray photoelectron spectroscopy analysis which was carried out on an ESCALAB 250 X-ray photoelectron spectrometer (XPS, Thermo Scientific, USA). Nitrogen adsorption/desorption isotherms at 77 K were measured using a Micromeritics ASAP 2020 system. The specific surface areas and pore size distributions were calculated by utilizing the Brunauer-Emmett-Teller (BET) method and Barret-Joyer-Halenda (BJH) model, respectively.

### Construction of the LIBs and electrochemical measurement

The working electrodes were prepared by mixing the non-graphitizable PPy-derived carbon/MWCNTs or the non-graphitizable PPy-derived carbon with acetylene black and polyvinylidene fluoride in a weight ratio of 80:10:10 in N-methyl-2-pyrrolidone. The coin-type batteries (LIR2025) were assembled with lithium metal as the counter/reference electrode and 1 M LiPF_6_ in ethylene carbonate, diethyl carbonate and ethylmethyl carbonate (1:1:1 by volume) as the electrolyte. The automatic charge/discharge equipment (LAND CT2001A) was used to perform the galvanostatic discharge/charge tests at a current density of 100 mA g^−1^ in the voltage range 0.005–3.0 V at room temperature, and rate performance was also tested at different current densities in the same voltage range. Cyclic voltammogram (CV) measurements were performed on an electrochemical workstation (CHI650D, Shanghai Chenhua Instruments Ltd.) at a scan rate of 0.1 mV s^−1^ from 0.005 to 3.0 V at room temperature.

## Additional Information

**How to cite this article**: Jin, B. *et al*. Facile Synthesis of Non-Graphitizable Polypyrrole-Derived Carbon/Carbon Nanotubes for Lithium-ion Batteries. *Sci. Rep.* **6**, 19317; doi: 10.1038/srep19317 (2016).

## Figures and Tables

**Table 1:** Comparison of specific capacities of carbon reported in the literatures and in this work.

Electrode material	Current Density (mA g^−1^)	Cycle Number	Capacity (mAh g^−1^)	Reference
Non-graphitic PPy-based carbon nanotube	100	150	544.6	[14]
Carbon nanosphere	60	60	~400	[15]
Polysilazane/potato starch	36	400	434	[39]
Carbon microtube	25	20	443	[41]
Carbon nanofiber	100	550	~460	[43]
N-doped fused carbon fiber	30	50	~550	[44]
N-doped carbon nanofiber web	100	10	~605	[45]
Nano-sized hard carbon spherule	20	13	525	[46]
Carbon monolith	74.4	40	~500	[48]
TNGC/MWCNTs	100	100	561	This study
